# Epstein-Barr Virus Association with Peptic Ulcer Disease

**DOI:** 10.1155/2015/164840

**Published:** 2015-06-24

**Authors:** María G. Cárdenas-Mondragón, Javier Torres, Lourdes Flores-Luna, Ricardo Carreón-Talavera, Margarita Camorlinga-Ponce, Ezequiel M. Fuentes-Pananá

**Affiliations:** ^1^Unidad de Investigación Médica en Enfermedades Infecciosas y Parasitarias (UIMEIP), Hospital de Pediatría, CMN Siglo-XXI, Instituto Mexicano del Seguro Social (IMSS), Avenida Cuauhtémoc 330, Colonia Doctores, Delegación Cuauhtémoc, 06720 Ciudad de México, DF, Mexico; ^2^Centro de Investigación en Salud Poblacional, Instituto Nacional de Salud Pública, Avenida Universidad 655, Colonia Santa María Ahuacatitlán, 62100 Cuernavaca, MOR, Mexico; ^3^Unidad de Investigación en Virología y Cáncer, Hospital Infantil de México Federico Gómez, Dr. Márquez 162, Colonia Doctores, Delegación Cuauhtémoc, 06720 Ciudad de México, DF, Mexico

## Abstract

*Background*. *Helicobacter pylori* (*HP*) infection and nonsteroidal anti-inflammatory drugs (NSAID) use are considered the main risk to develop peptic ulcer disease (PUD). However, PUD also occurs in the absence of *HP* infection and/or NSAID use. Recently, we have found evidence that Epstein-Barr virus (EBV) reactivation increases the risk to develop premalignant and malignant gastric lesions. *Objective*. To study a possible association between EBV and PUD. *Methods*. Antibodies against an EBV reactivation antigen, *HP*, and the *HP* virulence factor CagA were measured in sera from 207 Mexican subjects, controls (healthy individuals, *n* = 129), and PUD patients (*n* = 78, 58 duodenal and 20 gastric ulcers). Statistical associations were estimated. *Results*. Duodenal PUD was significantly associated with high anti-EBV IgG titers (*p* = 0.022, OR = 2.5), while anti-EBV IgA was positively associated with gastric PUD (*p* = 0.002, OR = 10.1). *Conclusions*. Our study suggests that EBV reactivation in gastric and duodenal epithelium increases the risk to develop PUD.

## 1. Introduction

Peptic ulcer disease (PUD) is a very common disease worldwide. It is characterized by lesions in the lining of the upper gastrointestinal tract, which often compromise all layers of the mucosa piercing the tissue and provoking bleeding. PUD most often presents as gastric or duodenal. Both forms of PUD share the same causative factors, mainly* Helicobacter pylori* (*HP*) infection and long-term nonsteroidal anti-inflammatory drugs (NSAID) use, which cause increased acid secretion and gastric inflammation. Still, there are important differences between gastric and duodenal ulcers; only gastric ulcers are considered a risk factor for gastric cancer (GC), and it is estimated that about 2% of patients with GC presented evidence of gastric ulcer [1–4].

PUD also occurs in the absence of* HP* infection and/or NSAID use (often referred to as idiopathic PUD), with reports supporting a prevalence of 20–40% of idiopathic PUDs in North America [5, 6] and of up to 40% in Asia [7]. PUD often recurs after* HP* pharmacological elimination [8]. All these data together support additional causes of PUD. More recently, Epstein-Barr virus (EBV) infection has also been linked to GC and early inflammatory lesion leading to GC [9–16]. The role of EBV in PUD has been poorly studied, with only two reports addressing an association between EBV and this disease [17, 18]. Both studies found EBV DNA positivity (by qPCR) preferentially associated with PUD when compared to tissues from individuals without disease. None of these studies addressed EBV serology.

*HP* is considered a cancer-inducing agent through chronic inflammation/tissue damage mechanisms. More recently, the bacterial virulence factor CagA has been documented as a classical oncogene [19], and* HP* cagA+ strains are associated with an increased risk of PUD [20, 21]. EBV infection has been associated with several types of B cell lymphomas and upper digestive tract carcinomas. We have recently documented an association between EBV reactivation antibodies and severe inflammatory responses in the gastric mucosa of pediatric and adult patients with gastric disease (from nonatrophic gastritis to cancer) [22, 23]. Taken together, all these findings support a critical EBV activity in promoting inflammation and disease of the gastrointestinal (GI) mucosa. In this study, we present serological evidence suggesting that EBV reactivation increases the risk to develop PUD.

## 2. Materials and Methods

### 2.1. Study Population

The study included 78 adult patients (≥30 years old) with any type of PUD. Patients were recruited between October 1999 and July 2002 after attending the Gastroenterology Units of the participant hospitals because of gastroduodenal symptoms. Healthy blood donors (the HI control group) were recruited between September 2010 and April 2012 from the Blood Bank of the Centro Medico Nacional Siglo XXI (IMSS).

### 2.2. Ethics Statement

The Scientific and Ethics Committees from each of the participating hospitals approved this study: Hospital de Especialidades (Instituto Mexicano del Seguro Social; IMSS), Gabriel Mancera (IMSS), Hospital General de México “Eduardo Liceaga” (Secretaría de Salud), all these hospitals in Mexico City, and the Blood Bank of the Centro Medico Nacional Siglo XXI-IMSS in Mexico City. All patients and healthy individuals (HI) were informed on the nature of the study and those willing to participate signed a written informed consent prior to specimen collection.

### 2.3. Study Design

This is a case-control study of patients with PUD in which antibodies against an EBV reactivation antigen,* HP*, and the CagA virulence factor were analyzed for association with this type of lesion. For all analysis, ulcer lesions (cases) were compared against HI (controls).

### 2.4. Data Collected

Sociodemographic data and clinical information were registered in questionnaires at time of inclusion. The information collected included age, gender, clinical symptoms, and clinical diagnosis based on endoscopy, histology, and clinical presentation. Patients with antibiotic, bismuth compounds, proton pump inhibitors, and/or nonsteroidal anti-inflammatory drugs or antiacid treatments three weeks prior to sample collection were excluded from the study.

### 2.5. Clinical and Histopathological Diagnosis

Because obtaining tissue samples of PUD lesions presents a high risk of bleeding, PUD diagnosis was based on endoscopy findings. Different biopsies from the stomach were taken to address gastric inflammation and other gastric lesions such as gastritis, intestinal metaplasia, dysplasia, or cancer. Three biopsies from the gastric antrum and three from the body were used for the histopathological diagnosis. All biopsies were fixed in formalin and embedded in paraffin, and a section was stained with hematoxylin and eosin (HE). HE stained sections were used to measure and classify the inflammatory reaction according to the updated Sydney system [24]. A pathologist expert analyzed all samples after standardization of the criteria using consensus protocol reading [25].

### 2.6. Collection of Blood

A sample of venous blood (4 mL) was drawn from all patients. Stored serum samples were used to analyze IgG and IgA antibodies against EBV viral capsid antigen (VCA), as well as IgG antibodies against* HP* whole-cell extracts and CagA protein by enzyme-linked immunosorbent assays (ELISA).

### 2.7. Determination of Anti-EBV VCA Antibodies

Anti-EBV VCA antibodies were determined using ELISA commercial kits (HUMAN; Wiesbaden, Germany), for IgG anti-VCA (catalog 51204) and for IgM anti-VCA (catalog 51104), as well as IgA anti-VCA (Diagnostic Automation, Inc., CA, catalog 1414-11) following manufacturer instructions and as previously described [23]. The reported value is the average of two independent assays. A subgroup of samples was done in quadruplicate using different lots of the ELISA kits to check for reproducibility. Calculations for antibody titers were done according to the manufacturer's instructions and the values are reported as HU units/mL for IgG.

### 2.8. Determination of Antibodies Anti-*H. pylori* and Anti-CagA

IgG antibodies against* HP* and CagA were determined using ELISA tests previously used and validated in a Mexican population [23, 26]. Patients were considered positive for* HP* antibodies when ELISA units were ≥1.0 and for CagA when ELISA units were ≥1.5, according to the validated cut-offs [26].

### 2.9. Statistical Analysis

The dataset was analyzed using different statistical tests. For continuous variables with normal distribution, the mean and standard deviation were used; if the variable was not normal, the median and range were used. Nonnormally distributed variables (antibody titers) were analyzed by the Kruskal-Wallis followed by the Mann-Whitney *U* tests. A one-way analysis of variance (ANOVA) followed by Student's *t*-test was used to address age differences. Associations between the type of PUD and the frequency of EBV and* HP* positives were estimated using odds ratios (ORs) with 95% confidence intervals (CIs). ORs were also used to estimate whether increased anti-EBV IgG titers were associated with duodenal PUD. For this analysis, the EBV IgG titer was categorized by tertiles based on their distribution in the HI control group, followed by a comparison of the highest to the lowest tertiles. A test for trend was used to analyze whether increased IgG titers correlated with increased ORs. Because sex and age are confounders, ORs were adjusted by them using logistic regression. A robust linear regression model was also used to adjust the variance of the antibody titers. Despite the size, the model showed a significant explanatory power with a *R*-squared of 51.5% for antibodies anti-EBV IgA in gastric PUD and of 20.5% for IgG in duodenal PUD. Statistical significance was set up at *p* ≤ 0.05. Data were analyzed using the statistical Stata 13.0 software program (Stata Corporation, College Station, TX, USA).

## 3. Results

### 3.1. Study Population

The study included 78 adult patients with PUD: 58 with duodenal and 20 with gastric ulcers and 129 HI controls. Age and gender of patients and controls, as well as the seroprevalence of anti-EBV, anti-*HP*, and anti-CagA antibodies, are summarized in Table 1. The HI group was significantly younger than patients with PUD. All subsequent statistical analyses were adjusted by age and gender.

### 3.2. Increased Levels of Anti-EBV IgG Antibodies Were Significantly Associated with Duodenal Ulcer Disease

To address whether EBV reactivation correlates with PUD, IgG and IgA antibodies against the lytic viral capsid antigen (VCA) were measured in all patients and controls (Table 1). Although almost all subjects included in the study were positive for IgG anti-EBV antibodies, the median of the antibody titer was significantly higher in patients with duodenal ulcers (89.2 HU/mL) than in gastric ulcer patients (73.9 HU/mL) and HI controls (73.7 HU/mL). On the other hand, IgA antibodies showed a significant increased frequency of positives between gastric ulcer patients (70%) compared to HI controls (17.1%). No differences were found between duodenal ulcer patients (31%) and controls. Regarding* HP* infection, the frequency of duodenal ulcer patients seropositive for* HP* (93.1%) and CagA (74.1%) was significantly higher than the HI controls (60.5 and 43.4%, resp.). The titer of anti-*HP* IgG was also significantly higher in the duodenal ulcer group.

### 3.3. EBV Seropositivity Increases the Risk to Develop Both Gastric and Duodenal Types of Peptic Ulcer Disease

Because most subjects were IgG positive for EBV, we tested whether increased IgG titers against EBV-VCA were at increased risk to develop PUD (Table 2). For this analysis, the healthy control group was divided into tertiles according to the titer of antibody, and comparisons were made between the frequencies of PUD patients in each preestablished tertile. This analysis showed that duodenal ulcer patients with high anti-EBV IgG have a significant OR [2.5 (1.04–5.8)] suggesting increased risk. Duodenal ulcer patients also showed a significant trend when progressing from low to medium to high IgG titers (*p* for trend = 0.022). In contrast, gastric ulcer patients did not show any significant association with anti-EBV IgG. We have previously analyzed pediatric and adult gastric lesions along Correa's sequence (atrophic gastritis to intestinal type GC) finding that anti-EBV antibodies also correlated with the level of immune cell infiltration in the lesion [22]. A similar analysis in the duodenal PUD patients did not show any correlation between EBV and the level of inflammation (Supplementary Table 1 in Supplementary Material available online at http://dx.doi.org/10.1155/2015/164840). This analysis showed that the majority of the duodenal ulcer patients presented moderate levels of immune cell infiltration.

The frequency of IgA anti-EBV positives was also used to estimate ORs comparing each disease group against the HI controls (Table 3). A significant OR was observed for EBV IgA and gastric ulcer patients (OR = 10.1 [2.4–43.2]). Contrary to the positive associations observed with EBV IgG, the IgA-based OR for duodenal ulcer patients was not significant (OR = 2 [0.9–4.7]). 16 patients (8 of gastric and 8 of duodenal ulcers) presented an additional gastric lesion (15 intestinal metaplasia and one atrophic gastritis). To test whether the EBV serology was not influenced by lesions different than PUD, ORs and trends were estimated with the patients that solely presented PUD (Supplementary Table 2). Although the numbers obtained were slightly different, all EBV significant associations were maintained, supporting the link between EBV and PUD.

A similar analysis was carried out with anti-*HP* and anti-CagA IgG antibodies finding significant ORs for duodenal PUD patients but not for gastric PUD patients (data not shown). The results for anti-*HP* and anti-CagA are in line with previous studies with larger sample sizes and part of these results (*HP* serology) were published previously [27].

## 4. Discussion

Gastric and duodenal ulcers are GI inflammatory diseases differentiated by their location and by the risk they confer to develop GC. Although it is believed that* HP* infection is the main risk factor for both types of ulcers, duodenal ulcers have not been associated with GC. EBV infection has also been associated with GC but a possible viral role in PUD has been poorly studied. Shukla et al. [18] found a positive association (*p* < 0.001) between the presence of EBV genomic sequences in tissues from PUD patients (70%) when compared to nonulcer dyspepsia tissues (37%). Similarly, Saxena et al. [17] found a positive association (*p* < 0.001) between EBV and PUD (75.5%) compared to nonulcer dyspepsia (37.3%). The latter study also reported an association between EBV and* HP* in the PUD pathogenesis, although neither study distinguished between gastric and duodenal ulcers. EBV infection of healthy duodenal epithelial cells was also reported in another study [28].

In this study, we provide serological evidence of an association between EBV infection and PUD. We found that EBV presents two different responses for each PUD, while IgG anti-EBV titers were significantly elevated in duodenal ulcers; in gastric ulcer patients a positive association was observed only with IgA antibodies. These data suggest that whereas development of duodenal ulcers might be influenced by the EBV systemic infection, gastric ulcers seem to be better associated with a local EBV effect. We recently analyzed a series of gastric lesions [22] in which we observed that the frequency and the titer of anti-EBV IgG were significantly associated with GC precursor lesions (atrophic gastritis, intestinal metaplasia, and dysplasia) and with the intestinal type of GC, while no association was found with anti-EBV IgA. In a similar study, individuals with GC precursor lesions (mainly intestinal metaplasia and dysplasia) were found positively associated with anti-EBV IgG, but not with anti-EBV IgA [29]. It is not clear why local gastric EBV reactivation, triggering IgA responses, may not be linked to gastric cancer or its precursor lesions but it may be associated with gastric ulcers. It has been suggested that patients with gastric ulcer are at increased risk of gastric cancer, although this has not yet been confirmed; one possibility is that gastric ulcer is a disease unrelated to gastric cancer, as it has been documented for duodenal ulcers.

EBV serology has been tested in multiple association studies, with the most consistent link found for anti-EBV IgA and nasopharyngeal carcinoma (NPC). In studies carried out in NPC endemic areas, the titer of anti-EBV IgA targeting reactivation antigens (such as VCA) has been shown to correlate with an increased risk of progression to NPC [30–33] and reviewed in [34]. Hence, anti-EBV IgA screening is being pursued in regions of high NPC incidence to identify individuals at risk, aiming to counteract the NPC-related mortality [35]. On the contrary, IgA levels are often not elevated in lymphoma patients, and anti-EBV IgG measurements have been the preferred choice in epidemiologic studies of EBV associated lymphomas [36, 37] (reviewed in [34] and references therein). The cause and clinical significance of these differential associations between disease and either anti-EBV IgA or IgG are presently not understood. Still, all these studies highlight the strength of the associations between anti-EBV antibodies and the risk for an EBV associated malignancy. Also, differences in types of the elevated antibodies have served to propose different mechanisms of viral carcinogenesis and biomarkers for risk stratification.

Regardless of the type of systemic or local viral mechanisms, these findings suggest that there is a host inability to control EBV infection, marked by the elevation of antibodies against viral reactivation antigens, increasing the risk of developing duodenal or gastric ulcers. Surprisingly, we did not find any association between EBV and inflammation. An important difference when taking samples from PUD patients is that biopsies cannot be obtained directly from the lesion due to the risk of bleeding. This lack of correlation may be because the biopsies used for this study are of nonulcerative tissue. The analysis of a larger set of samples is necessary to confirm this and previous studies linking EBV with PUD.

We have previously documented that patients coinfected with EBV and* HP* have an increased risk to develop severe gastric inflammation, gastric premalignant lesions, and cancer [22, 23]. In this study,* HP* serology was also positively associated with duodenal ulcers suggesting that both pathogens cooperate to increase the risk to develop PUD. Unfortunately, the number of samples in the present study prevented further stratifications to compare the PUD risk between double versus single infected patients. How EBV and* HP* interact at the tissue or cellular level needs to be addressed in subsequent studies. Also, we did not have tissue to analyze whether EBV resides in B cells or epithelial cells in the lesion and whether there is an important lytic activity, as the increased anti-VCA antibodies suggest. In our previous study, we found very few adults positive to anti-VCA IgM, and IgM positivity correlated with mild gastritis [22]. On the other hand, testing for latent viral genes, such as EBNA-1, would be informative about whether there is an increase in viral latency parallel to the increase in viral reactivation. However, the importance of the latter is that it implies that PUD patients could benefit from drugs targeting the EBV viral lytic cycle, while there are currently no drugs targeting EBV latency.

## Supplementary Material

Supplementary table 1 shows that there is not association between the level of immune cell infiltration (mononuclear and polymorphonuclear cells) in the ulcerative lesion and the level of IgG anti-EBV antibodies. Most duodenal ulcer patients presented moderate levels of immune cell infiltration.
Supplementary table 2 shows ORs and trends of patients that solely presented PUD. For this analysis patients with intestinal metaplasia (N=15) and atrophic gastritis (N=1) were eliminated, confirming the association between EBV serology and ulcer.

## Figures and Tables

**Table 1:** General description of the study population.

Variable	Healthy controls^a^	Peptic ulcer	
		Duodenal	Gastric
*N* total (%)	129 (100)	58 (100)	20 (100)
Age (mean ± SD)	41.6 ± 7.7	51.7 ± 12.3^**∗∗**^	63.7 ± 18.8^**∗∗**^
Sex, male : female ratio	64/65 = 0.98	31/27 = 1.15	10/10 = 1

EBV positives, IgG *n* (%)	128 (99.2)	58 (100)	20 (100)
Anti-IgG EBV^b^ median	73.7	89.2^**∗∗**^	73.9
Anti-IgG EBV (range)	(20.1–160.7)	(21.2–182.2)	(25.2–183.5)
EBV positives, IgA *n* (%)	22 (17.1)	18 (31.0)	**14 **(70)^**∗∗**^

*HP* positives, *n* (%)	77 (60.5)	**54 **(93.1)^**∗∗**^	14 (70)
*Anti-HP* ^c^ median	2.5	5^**∗∗**^	1.9
*HP* (range)	(1–10.9)	(1–16.2)	(1–7.8)
CagA positives, *n* (%)	52 (43.4)	**43 **(74.1)^**∗**^	9 (45)
Anti-CagA^c^ median	4.9	5.5	3.9
CagA (range)	(1.7–26.1)	(1.7–23.6)	(2–8)

Significant differences: ^**∗**^ *p* ≤ 0.05 and ^**∗∗**^ *p* ≤ 0.001. SD: standard deviation.

^a^Used as control group for comparison.

^b^Units (HU/mL).

^c^ELISA units.

**Table 2:** Odds ratios and trends for EBV IgG antibody titers and peptic ulcer disease.

EBV IgG titers^b^	Healthy controls^a^	Peptic ulcer			
		Duodenal		Gastric	
	*n*	*n*	OR (95% CI)^c^	*n*	OR (95% CI)^c^
20.1–55.09	42	14	1.0	6	1.0
55.10–83.76	43	11	0.9 (0.4–2.5)	6	0.9 (0.3–3.4)
83.77–181.1	43	33	**2.5 **(1.06–5.7)^**∗**^	8	1.5 (0.4–5.6)
*p* for trend^d^			0.022^**∗**^		0.529

Significant differences: ^**∗**^ *p* ≤ 0.05.

^a^Used as control group.

^b^Units (HU/mL).

^c^OR and ^d^Chi square for trend, adjusted for age and sex and also adjusted by a robust logistic regression model.

**Table 3:** Odds ratios for EBV or *H. pylori* infection and peptic ulcer disease.

Serology	Healthy controls^a^	Peptic ulcer	
		Duodenal	Gastric
*N* (%)	129 (100)	58 (100)	20 (100)
EBV IgA			
Positives, *n* (%)	22 (17.1)	18 (31)	**14 **(70)^**∗∗**^
Negatives, *n* (%)	107 (82.9)	40 (69)	**6 (30)**
OR^b^ (95% CI)		2.0 (0.9–4.6)	**10.1 (3.0–33.7)**
*p* ^c^		*p* = 0.089	**p** = 0.0001
*HP *IgG			
Positives, *n* (%)	77 (59.7)	54 (93.1)	14 (70)
Negatives, *n* (%)	52 (40.3)	4 (6.9)	6 (30)
OR (95% CI)		**7.2 (2.8–20.8)**	0.8 (0.3–2.7)
*p* ^c^		**p** = 0.0001	*p* = 0.743
*HPCagA*+ IgG			
Positives, *n* (%)	52 (40.3)	43 (74.1)	9 (45)
Negatives, *n* (%)	77 (59.7)	15 (25.9)	11 (55)
OR^b^ (95% CI)		**3.8 (1.8–8.0)**	0.8 (0.3–2.4)
*p* ^c^		**p** = 0.0001	*p* = 0.714

Significant differences: ^**∗∗**^ *p* ≤ 0.001.

^a^Used as control group.

^b^OR and ^c^Chi square for trend adjusted for age and sex and also adjusted by a robust logistic regression model.
