# Supplementary material for: Epstein-Barr Virus Association with Peptic Ulcer Disease
Source: Anal Cell Pathol (Amst). 2015 Jun 24;2015:164840. doi: 10.1155/2015/164840 (PMC4495172; doi:10.1155/2015/164840)
Supplement: Supplementary file 1 — Supplementary table 1 shows that there is not association between the level of immune cell infiltration (mononuclear and polymorphonuclear cells) in the ulcerative lesion and the level of IgG anti-EBV antibodies. Most duodenal ulcer patients presented moderate levels of immune cell infiltration. Supplementary table 2 shows ORs and trends of patients that solely presented PUD. For this analysis patients with intestinal metaplasia (N=15) and atrophic gastritis (N=1) were eliminated, confirming the association between EBV serology and ulcer. [file 164840.f1.pdf]

Supplementary tables

Supplementary table 1. Analysis of anti-EBV IgG and inflammation.

EBV IgG titers[b]	Mononuclear cell infiltrate		
	Mild-Moderate[a]	Severe	OR (95% CI)[c]
	n	n	
20.1 – 55.09	8	6	1.0
55.10 – 83.76	5	6	1.3 (0.2 – 7-3)
83.77 – 181.1	23	10	0.5 (0.1 – 2.0)
p for trend[d]			0.226
**EBV IgG titers[b]**	**Polymorphonuclear cell infiltrate**		
	**Mild-Moderate[a]**	**Severe**	OR (95% CI)[c]
	n	n	
20.1 – 55.09	12	2	1.0
55.10 – 83.76	10	1	0.5 (0.03 – 7.1)
83.77 – 181.1	28	5	1.2 (0.2 – 8.9)
p for trend[d]			0.75

[a] Used as control group.
[b] Units (HU/mL)
[c] OR and [d] Chi square for trend, adjusted for age and sex. Also adjusted by a robust logistic regression model.

Supplementary table 2. ORs estimated after eliminating patients with intestinal metaplasia.

**Serology**	**Healthy controls**[a]	**Peptic ulcer**	
		**Duodenal**	**Gastric**
N (%)	129 (100)	50 (100)	**12 (100)**
EBV IgA			
Positives, n (%)	22 (17.1)	17 (34)	**8 (66.7)****
Negatives, n (%)	107 (82.9)	33 (66)	**4 (33.3)**
OR[b] (95% CI)		**2.3 (1.01-5.3)**<br>**p = 0.048**	**8.4 (2.0-35.4)**<br>**p = 0.003**
HP IgG			
Positives, n (%)	77 (59.7)	48 (96)	7 (58.7)
Negatives, n (%)	52 (40.3)	2 (4)	5 (41.3)
OR (95% CI)		**11.5 (2.6-50.8)**<br>**p = 0.001**	0.4 (0.1-1.8)<br>p = 0.216
HPCagA+ IgG			
Positives, n (%)	52 (40.3)	**38 (76)**	3 (25)
Negatives, n (%)	77 (59.7)	**12 (24)**	9 (75)
OR[b] (95% CI)		**4.1 (1.9-8.9)**<br>**p = 0.0001**	0.3 (0.08-1.1)<br>p = 0.071

Significant differences *****($p \leq 0.05$) and ******($p \leq 0.001$).
[a] Used as control group.
[b] OR adjusted for age, sex. Also adjusted by a robust logistic regression model.
